# ‘Between Inner Strength and Fighting Prejudice’: Psycho-Social Processes Implemented by Women with Leukemia Along the Illness Trajectory: A Grounded Theory Study

**DOI:** 10.3390/curroncol31100468

**Published:** 2024-10-18

**Authors:** Giovanna Artioli, Chiara Taffurelli, Victoria Cervantes Camacho, Stefano Botti, Roberto Lupo, Luana Conte, Paola Ferri, Antonio Bonacaro

**Affiliations:** 1 Department of Medicine and Surgey, University of Parma, 43121 Parma, Italy; chiara.taffurelli@unipr.it (C.T.); victoriacervantes.camacho@unipr.it (V.C.C.); antonio.bonacaro@unipr.it (A.B.); 2 Department of Haematology, Azienda USL-IRCCS of Reggio Emilia, 42122 Reggio Emilia, Italy; stefano.botti@ausl.re.it; 3 “San Giuseppe da Copertino” Hospital, Local Health Authority (ASL) of Lecce, 73043 Copertino, Italy; roberto.lupo@asl.lecce.it; 4 Department of Physics and Chemistry, University of Palermo, 90128 Palermo, Italy; luana.conte@unipa.it; 5 Department of Biomedical, Metabolic and Neuroscience Sciences, University of Modena and Reggio Emilia, 41121 Modena, Italy; paola.ferri@unimore.it

**Keywords:** leukemia, women, gender medicine, illness trajectory, women’s perceptions, psycho-social process, grounded theory

## Abstract

Background: Disease trajectories in leukemia are often unpredictable and recurrent, and patients’ experiences can impact their quality of life. Studies in the literature often do not explore gender-related illness experiences from an intersectional approach and throughout the illness trajectory. This comprehensive study aims to explore the full spectrum of experiences lived by women with leukemia throughout the disease trajectory, from diagnosis to treatments and post-stem cell transplant follow-up. Method: A grounded theory approach was meticulously developed to analyze semi-structured interviews with 13 women with leukemia in the post-transplant follow-up phase at a hospital in Northern Italy. The data analysis was an iterative process, conducted concurrently using a constant comparative method. Data collection concluded when data saturation was reached. Results: The core category identified is women’s inner strength during the disease trajectory, which was identified for its recurrence and cross-cutting nature, according to the women. This core category interconnects with five main categories: 1. Facing the disease: Between resistance and surrender. 2. Living for today and moving forward. 3. Unexpected elements in relationships. 4. Changes that shape women. 5. Demystifying the body and embracing ‘diminished beauty’. Conclusions: An explanatory model of the disease trajectory of women with leukemia was defined as: ‘Women with leukemia, between inner strength and fighting prejudice’. An in-depth analysis of the disease experiences revealed aspects that are not easily understood through a purely biological perspective of gender differences, often overlooking the psycho-social and relational peculiarities of women.

## 1. Introduction

Western medicine is beginning to realize that, especially for some diseases, the clinical and biological model can no longer respond effectively to the increasingly complex problems that people present [1].

This study pays attention to the dimensions of sex, gender, and intersectionality in leukemia, trying to capture how these constructs interact in health outcomes, research, and especially people’s perceptions of the disease.

The difference between sex and gender: ‘Sex’ and ‘gender’ refer to two concepts that are often confused but have clear distinctions in contemporary science. Sex refers to the biological and physiological characteristics of individuals, such as chromosomes, hormones, and reproductive organs, which generally categorize humans as male or female. In contrast, gender is a social and cultural construct that relates to the expectations, roles, and behaviors associated with being a man or a woman in a given society. Gender includes psychological and cultural aspects and can vary significantly across societies and historical periods. Moreover, gender is now recognized as non-binary, meaning there can be a diversity of gender identities beyond male and female [2].

The term sex, while referring to the biological dimension of the individual, is often used as a synonym for the term gender [3].

Several studies highlight the lack of specific literature on gender differences in medical treatment, underlining the need for further research. Nielsen [4] found that despite the increase in female participation in clinical research, gender differences are still under-researched and often overlooked in medical treatment protocols. Peters et al. [5] propose a roadmap to disaggregate health research by sex and gender, highlighting how the lack of gender-specific data is a barrier to improving health outcomes for women. Finally, a survey conducted by Paulsen [6] found that women often perceive a gender bias in the care they receive, with a significant proportion of women reporting feeling ignored or not taken seriously by their healthcare providers.

When it comes to acute and chronic pain, women may be victims of prejudice from caregivers and society [1,7] and may experience stigma and marginalization [8], leading to what is called ‘gender pain exaggeration bias’ [9].

In cardiovascular disease, contributions to gender-related differences are recognized but gender-related differences are still little explored [10].

The literature relates adverse events associated with drugs to gender [11,12], as well as the relationship between gender and the complex pattern of interaction between symptoms of depression and anxiety and physical, cognitive, and social factors [13,14].

Also, in oncology and oncohematology, the terms sex and gender are mainly used as synonyms and highlight the relationship between the biological dimension of sex and the disease condition [15,16].

Leukemia accounts for a significant proportion of hematological cancers but tends to affect women less frequently than men [17]. Women generally show a better prognosis and more prolonged survival than male patients, a phenomenon attributed to various biological, hormonal, and immunological factors [18]. Estrogens may play a protective role, modulating disease progression and improving response to treatment [19].

The distinction between sex and gender becomes even more significant when considering the concept of intersectionality. Intersectionality, a term coined by Crenshaw [20], refers to how various social identities—such as race, class, gender, sexual orientation, and others—interact and intersect to create unique experiences of discrimination or privilege. Understanding sex as a biological concept and gender as a social construct allows us to see how these categories do not exist in isolation. Instead, they intersect with other identities, leading to complex and varied experiences for individuals.

Grasping the differences between sex, gender, and intersectionality is critical in ensuring nuanced, inclusive approaches in research, policy, and healthcare. By understanding how these identities overlap and shape individuals’ realities, we can better address social inequalities and improve outcomes across different sectors of society [21,22].

Intersectionality becomes a theoretical approach that pays explicit attention to differences between groups and seeks to illuminate various interacting social factors that influence human life, including social positions, health status, and quality of life [23,24].

Intersectionality became essential to analyze the multiple modes of existence based on social, cultural, economic, and political processes that impact a person’s health [25].

Based on the concept of intersectionality, in leukemia, the most common symptoms (nausea, vomiting, mouth sores, but also fatigue, fever, weakness, bleeding, diarrhea, and infections) can have a negative impact on a patient’s quality of life, in particular, due to the side effects of treatments and the duration of treatment. Unpredictable and relapsing courses characterize these diseases, and patients’ opinions may vary along the disease continuum. Emotional experiences (anxiety, depression, fear of disease progression) reduce personal well-being, social situations, independence, interpersonal relationships, and cognitive disturbances [26,27]. Last but not least, there is also a financial burden related to costly and prolonged care that may impact clinical management, quality of life, and patient survival [28].

Intersectionality in health identifies certain factors, such as body image, family dynamics, and emotional resilience, that intersect with each other and other factors to create unique challenges. It also identifies varied experiences of illness.

This approach becomes crucial to overcome the limitations of traditional gender-only analyses [24,29].

Body image modification is one of the main problems for leukemia patients, especially after chemotherapy and bone marrow transplantation [30,31,32]. Women experience this problem, which is also linked to depression, decreased self-esteem, and poor quality of life [33].

Family relationships are also tested in oncohematological disease. A recent study showed that factors that promote family resilience, such as organizational flexibility, clear communication, and social support, also exist. At the same time, risk factors such as symptom burden, self-concealment, and social alienation exist [34].

Resilience emerges as a pivotal intersectional element in the context of cancer and oncohematology. Its close relationship with the quality of life and mental health outcomes of the ill is a significant finding [35]. Resilient individuals demonstrate better coping abilities and fewer problems than their less resilient counterparts. Psychological and social factors play a key role in fostering resilience, which can be viewed as a protective factor for these individuals [36]. However, it is important to note that women may feel pressured to ‘be strong’ and downplay their emotional or physical struggles to meet societal expectations of resilience, all while managing the impacts of the illness on their self-esteem and relationships.

In this intersectional view, the experience of leukemia in women is not only about the disease but also about how the disease interacts with social norms regarding beauty, resilience, and emotional strength, creating a distinct and multifaceted experience.

There is still limited evidence on the experiences of leukemia patients along the disease pathway [37]. In most cases, these are qualitative studies exploring patients’ experiences at specific stages of the disease: before treatments [38], during treatments [39], in the advanced stages of the disease [40], or the last year of life [41]. These results appeared fractional along patients’ disease trajectories without showing gender-related differences.

To our knowledge, there are no studies available in the literature on the multidimensional and multifaceted experiences of women with leukemia during the disease.

This study aims to explore the experiences of women with leukemia along the disease’s trajectory, from diagnosis to treatment and follow-up after stem cell transplantation (SCT).

## 2. Materials and Methods

### 2.1. Study Design

The generative question of the study was: “What are the experiences lived by a woman diagnosed with leukemia along the disease pathway until the post-SCT phase”? This question presupposed the deepening of various biopsychosocial processes that were put in place by women faced with a highly complex problem: receiving the diagnosis of leukemia. The patient must be able to pass through all stages of this unpredictable disease, including SCT and post-transplant stage.

For this reason, the researchers chose the grounded theory (GT) method, proposed by Corbin and Strauss [42,43], as their research design.

GT is a qualitative research methodology that involves a systematic and, at the same time, flexible process for collecting and analyzing data simultaneously. This process leads to the elaboration of concepts or theories concerning a particular phenomenon, starting directly from data [44,45].

GT aims to help researchers understand the psycho-social dynamics that influence human behavior. Researchers use the approach to develop new theories in the absence or lack of evidence regarding certain circumstances [46].

In this study, the consolidated criteria for reporting qualitative studies (COREQ) 32-item checklist was followed for reporting results [47].

### 2.2. Research Setting

The research was carried out between October 2020 and February 2021 in an oncohematology department of a hospital located in Northern Italy. This department deals with diagnosing and treating oncohematological diseases, offering in- and out-patient services. The main areas of activity included the hemopoietic SCT, both autologous and allogeneic, and the diagnosis and treatment of acute leukemia and other malignant or non-malignant diseases.

### 2.3. Research Team

The research team was multi-professional, composed of nurses (GA, CT, RL, SB, AB, LC, PF) and psychologists (CT and VCC), experts in oncohematological disease (SB, RL, PF) and qualitative research (PF, AB, LC), and experts in grounded theory methodology (GA, CT, VCC).

### 2.4. Study Population and Recruitment

The study population consisted of women with leukemia in post-SCT FU. Participants were selected through a convenience sample.

The research team trained the managers and other collaborators of the involved structure about the study’s objectives and procedures. The local nurse involved in the research provided the principal investigator with a list of potential participants who met the inclusion criteria. Those were subsequently contacted by the research team to be informed about the study proposal and to plan the informed consent meeting and to agree on a date and place for the study interview.

#### Sampling

The study was only aimed at women who had leukemia and had passed through the whole disease path until the post-SCT period.

The women were all cisgender (people whose identity as a person coincides with the gender assigned at birth) white, born and living in Northern Italy, married or with a partner, women who expressed themselves with the results we herein report that are typical of a culture, in Italy, that is still prevalent. The health service they used is a public health service that covers all the costs of illness, especially cancer.

The interviews were carried out in the post-transplant FU to collect perceptions and experiences on the whole trajectory of the disease. The inclusion and exclusion criteria are presented in Table 1.

### 2.5. Ethical Statement

Before starting the interview, participants were asked to sign an informed consent form that included a study briefing note. The document also specified that the interview would be recorded and that the data collected would be analyzed and presented in aggregated form, ensuring complete anonymity of the participants. The study has received approval from the Area Vasta Emilia Nord Ethics Committee, Reggio Emilia, Protocol n° 2019/0085765–19 July 2019. The principles of the Helsinki Declaration informed the study.

### 2.6. Data Collection

Data collection was carried out through a semi-structured interview following the principles of grounded theory. It was carried out at an early stage to facilitate open coding. However, it was implemented using an iterative data collection and analysis approach: researchers continued to collect and analyze data as the theoretical model developed [42].

Field notes recorded by observers who had supported the interviewer during the interviews were also collected and included in the analysis.

In addition, several memos were developed, the result of repeated listening to interviews to compare them to generate new ideas in the research group [48,49].

The interview consisted of a series of questions that were asked to all participants, favoring any further information. To ensure the interview was successful, it was essential that the interviewer created an environment based on empathic listening and mutual trust. To reduce interviewer bias, all interviews were conducted by experienced research nurses (CT and GA) in a private space within the Oncohematology Unit, with the presence of an observer from the research group who took notes during and after the interview (VCC and SB).

Each interview was conducted face-to-face, lasted between 30 and 50 min, and was audio-recorded.

The audio recording was encoded and transcribed verbatim by a member of the research team and then analyzed.

The interview outline is shown in Table 2.

### 2.7. Data Analysis

Interview data were examined following the three phases and principles of Strauss and Glaser’s grounded theory (i.e., open, axial, and theoretical coding), using the constant comparative method [46,50,51]. In Table 3, we show the characteristics of the different types of coding.

Data from field notes during interviews and memos collected at different stages of data processing were also analyzed [54].

The data analysis was initially carried out by three researchers (G.A., C.T., V.C.C.) who, independently, analyzed the data and then compared their results to seek agreement. In cases of disagreement, a fourth investigator (S.B.) was involved. The analyses then continued with a continuous comparison in groups, elaborating memos and comparing data to reach ever-higher levels of conceptualization.

Data saturation occurred as predicted by Saunders et al. [54].

The process of writing memos is an important component in running a GT study. Writing memos allowed the researcher to go from the simplest description to richer and more mature memos, and finally to a rich and conceptual theory. Connections developed and became more conceptual during the entire memorization process [55]. The memos were compared with each other through a method called constant comparison. During the process of comparison and sorting, previously unknown connections were established [54]. The act of remembering promoted a deep dive into data, amplifying researchers’ abilities and receptivity to underlying connotations [56]. Memoing was used to assist the researchers in making conceptual leaps from raw data to those abstractions that explain research phenomena in the context in which they were examined. In Table 4 we summarize the process of processing data.

#### Rigor

Glaser and Strauss focused on credibility and validity to ensure the rigor of the methodology. They suggested criteria for assessing credibility, such as a detailed and vivid data description, so readers feel they have been in the field. They also proposed to use of more comparison groups to increase the scope and generality of the theory and to correct and adjust the emerging theory to different conditions [42].

## 3. Results

### 3.1. Study Participants

Combining the initial sampling and the theoretical sampling, a total of 13 women were interviewed, all in the post-transplant follow-up phase. Data saturation occurred at the twelfth interview and interview 13 confirmed saturation.

The women interviewed had an average age (±SD) of 46.5 (±7.8) years, the mean hospital stay during Stem Cell Transplantation (SCT) was 41.5 (±17.1) days and the mean times between diagnosis and interview and SCT and interview were 917.5 (±535.4) days and 638.2 (±446.1) days, respectively. None of the women interviewed had a history of mental health problems or sought assistance from the psychiatric service. The main characteristics of the study participants are described in Table 5.

### 3.2. Trajectory of Disease

The trajectory of disease for these women has always been uncertain and unpredictable. Once diagnosed, the women had to undergo several examinations to select more specific treatments. After the diagnoses were confirmed, we described the phase in circular mode. The illness trajectory was between the beginning of treatments, the remission phase, the possible exacerbation phase, and the initiation of new treatments, which may culminate in bone marrow transplantation. The post-transplant phase with possible complications and the control and follow-up phases followed. All women interviewed followed this disease trajectory until follow-up. The treatment plans were based on international guidelines specific to the disease staging of each malignant condition. In a general way, the pre-SCT clinical pathway of these conditions requires a number of sequential hospitalizations for high-dose chemotherapy and the management of its related side effects including pancytopenia, followed by home periods for patient health recovery. All recipients participating in this study had received induction therapy with or without consolidation treatment and allogeneic SCT, thus the graft matching with the patient caused to someone additional issues such as graft versus host disease (GvHD) and other engraftment-related complications such as virus reactivations. Our sample confirmed that adverse events and side effects due to chemotherapy toxicity such as febrile neutropenia (FN), oral mucositis (OM), and gastrointestinal problems including mucosal injuries, nausea, and vomiting are common conditions experienced by patients during hospital stay. However, GvHD, infections, sepsis, and specific organ insufficiency are dangerous conditions that can result in poor outcomes affecting post-SCT quality of life and/or impose on the patient in a life-threatening way. In Table 5 we present the characteristics of the participants during the treatment journey.

### 3.3. Psycho-Social Processes of Women in the Trajectory of Disease

**Figure 1:**
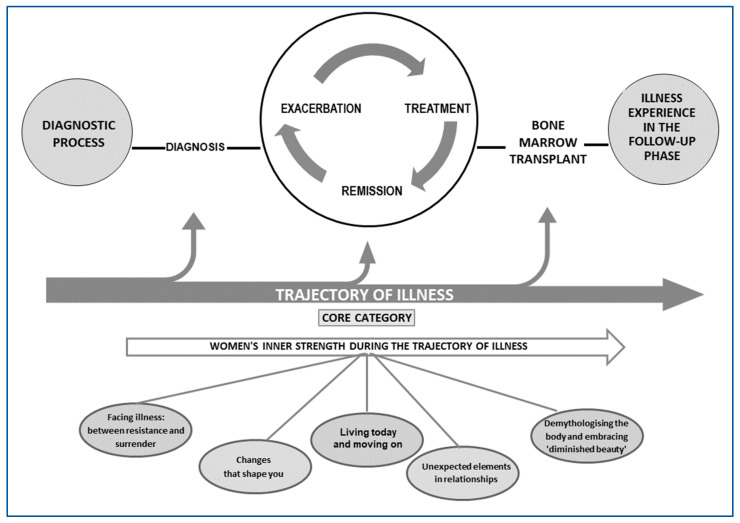
The theory: women with leukemia, between inner strength and fighting prejudice.

The psycho-social processes described by the women were closely correlated with their disease trajectory. We describe the main concepts identified below, which helped define a proto-model. From this, the research team, using memos, arrived at the definition of the core category and the main categories, developing the theoretical model presented in Figure 1, which provided answers to the study’s generative question.

#### 3.3.1. The Main Concepts

In the intermediate phase of the theorization, six core concepts were identified: strength, drama, fight, relationships, change, and resilience. Table 6 contains the main concepts of the theory elaborated through the codes and memos.

#### 3.3.2. The Theorization

The ‘core category’: “*Women’s inner strength during the illness trajectory*” is generated transversally from the data and was identified because of its recurrence but also due to the research team’s sensitivity in reading between the lines to identify a process particularly relevant to women. The core category is closely interconnected with five other main categories: *(i) Facing illness: between resistance and surrender; (ii) Living today and moving forward; (iii) Unexpected elements in relationships; (iv) Changes that shape Women; and (v) Demythologising the body and embracing ‘diminished beauty’.*

In Figure 1, we present the theory developed from the data.

##### The Core Category: Women’s Inner Strength during the Trajectory of Illness

The interviewees recognized that leukemia was initially presented as a drama that came suddenly to a clear sky, and the perception was that the world had collapsed. Then, slowly, they embarked on ‘the road to Calvary’. Women felt that they were isolated, that the disease was taking away their strength, and they became aware that the journey of illness would be long and very tiring. Women said:

“*And so no, it came like a thunderbolt in the clear sky*”.

(Cod. 1.33)

“*It was very hard (,.) but always with a positive vein*”.

(Cod. 6.14).

In this context, women’s inner strength emerged clearly—a force in which the interviewees themselves had difficulty believing or, conversely, had become aware of.

“*I did not think I had such a strong character*”.

(Cod. 3.191)

“*I was much stronger, I was already strong before, now I am much stronger, much more determined.*”.

(Cod. 2.346)

Some interviewees acknowledged that they already have strong personalities and were considered the pillars of their family. Others were surprised by the strength they had managed to find; some were amazed to be considered vital by others, also because they knew how much they had to struggle and suffer to face what happened to them. The women said:

“*[my husband] He keeps saying, ‘but you are so strong’, but they do not know that to become strong, the work I had to do, what I had to endure, how many times I cried for fear (…) always not to make it!*”

(Cod. 10.362)

It was an inner strength born from the ability to ask for help when they were in great difficulty, for example, asking for the help of a psychologist, and from the maturation of the awareness of being inevitably faced with gigantic things that were almost impossible to face.

The interviewees themselves sought this dimension of strength because they understood the importance of finding strength:

“*After I find the strength inside to overcome everything, I do not know how I do! Some say you have it or not; some say you make it come… I do not know… I am not afraid of anything and nobody!!! It is like this…. Maybe it is because we are women!*”

(Cod. 8. 537)

##### Main Categories

Category 1: Facing illness: between resistance and surrender.

Faced with the illness, the women assumed an attitude that we called ‘*between resistance and surrender*’, meaning that, from what they report, it appeared that they never really resisted the illness, nor did they ever fully surrender. Instead, they described behaviors of extreme involvement with the illness, but also of extreme attention to themselves, to the questions that came to them spontaneously, and to what was suggested to them by health professionals. Women said:

‘*I did not ask myself where I was going, I just followed the path*’

(Cod. 12.55).

“h*owever…you have to follow everything they tell you…you have to be very….. very meticulous in doing things…*”

(Cod. 5.105)

Faced with the disease and its questions, a relationship was created between the women and leukemia, where the keyword was ‘collaborate’. The women tried to cooperate without asking too many questions until the end. A woman affirmed:

“*Leukemia is a lady who has entered inside me…. I do not want to fight now, but I want to collaborate because she must understand that this is not her place because I have so many other things to do!…*”

(Cod. 10,162).

However, they were aware that there was no turning back and that at some point, in the face of this hard reality, they had to become aware that reality was what it was and that they had to deal with it.

Category 2: Living for today and moving forward.

The interviewees acknowledged that they had entered a different dimension from the previous one. If before, they took things for granted and made so many plans for the future, now the essential thing became to live for today and be happy to live again.

It became essential to stop for a moment to taste the significance of the moment, such as having a coffee, making meaningful even the tiny things, or doing something immediately that they would have previously postponed. The women said:

“*If one has the desire to go on a journey, to do strange things, that is not my case; you do not have to wait; you have to do it, and you are fine; if you have the chance, you have to do it*”

(Cod. 6.155).

“*I had been thinking about going paragliding for years, but I always put it off, but after this thing happened to me, I did not put it off any more; I do not put anything off any more.*”

(Cod. 12.255).

The second element the interviewees recognize as a critical condition of their illness is moving forward. In filigree, however, there was a strong thought, an act of will that the interviewees strongly desired to perform and that supported their difficult path of illness: go forward. The women relied on their present and their focus was on the next moment, so they said:

“*Slowly things will come, they will be different from those I had in mind, but we go on.*”

(Cod. 6.144).

“*I said: by now as it wants to go, it will go. But let us go ahead!…*”

(Cod. 10.27).

Category 3: Unexpected elements in relationships.

Relationships became relevant to the disease process of respondents. Women felt the need to share with others to understand that they were not alone in their illness. However, they realized that finding the right people was very difficult.

With this awareness, they took refuge in the most substantial ties, those of family, with particular attention to their husband or partner. Most of the interviewees acknowledged the availability of partners; however, sometimes misunderstandings arose due to the great desire of women “for presence”. In this context, they stated:

“*.. A person’s material acts do not count (…) what counts to me is being there in the bad times…*”

(Cod. 4.257).

During the illness, women encountered new and unpredictable situations, such as the fact that the family’s affections gradually became evident and constituted an essential point of support for them. They also highlighted new encounters, such as a new partner who was not known before but who, in the situation of illness, could stay close and be greatly attentive. Women also experienced unexpected elements of external relationships, such as friendships and new people who knew how to be close, like the most dear and familiar affections. Other relationships were also activated, such as those with the healthcare staff who showed unexpected tenderness towards patients. The interviewees expressed themselves as follows:

“*I felt part of a big family, which are these friends of mine who have always been there and new people a little of the family a little more in the strict sense family affections*”

(Cod. 11. 63).

Category 4: Changes that shape women.

In this new condition of a pause in a consolidated life of continuous discoveries or losses, women were at the mercy of various changes that touched and upset their certainties. These changes were very often physical but also of character and lifestyle, involving the deepest part of themselves, their identity, and their appearance, and having a “different eye” on their future, prospects, or expectations.

The disease left behind real traces, such as fatigue, that are perceived intensely. These changes continued, both at the physical level and at a more global level of the person, until they led to a profound and total change in the interviewees’ lives.

“*I fell into a deep crisis because my hair would not grow, they had a hard time recognising me and then having this chronic graft on my skin, it made me feel terrible for a year, and then big physical problems because I could not sleep at all*”

(Cod 8.206).

Another change that really upset the lives of the women, especially the younger ones, was the impossibility of having children after illness. The interviewees themselves said:

“*And children… children are the other big thing. I wanted children… I am not the kind of woman who wants to start a family the most, (…) but I am 35 years old, and I have never said I do not want children*”

(Cod. 3.180).

Some changes remained in the lives of the women, and that concerned ‘a different eye’ with which to look at the future, in the sense of living continuously in the fear that something unexpected will happen and you can start to feel bad again. In the same way, they lived constantly in a state of tension, waiting, and suffering for fear of never being able to reach their goal.

“*(…) I have pain in a finger (..) I always fear to have something different, again*.”

(Cod. 11.124)

Category 5: Demythologizing the body and embracing ‘diminished beauty’.

During the illness, the women activated a process of adaptation that led them at first to recognize, with regret and sorrow, that their bodies had changed, that they no longer recognized themselves, and did not like themselves in that body. So, they had trouble looking at themselves in the mirror. The women said:

“*When I looked at myself in the mirror and saw this swollen face, I said to myself I cannot go around and be seen like this with this face because I did not even have my features anymore*”

(Cod. 6.220).

In addition, the women were constantly confronted with the stereotype of female beauty and tried in every way to hide their ‘decrease in beauty’ even through small tricks such as putting on a wig or at least a headpiece, even at home, to go to bed.

The women had to face the fact that they were becoming the opposite of traditional beauty stereotypes and still felt themselves the object of external looks, which considered them different, and said:

“*You still feel the eyes on you that look a little ‘strange*”

(Cod. 5.124).

At the same time, however, the body was somewhat demystified, reintegrated into the new condition of ‘diminished beauty’ to leave room for elements of their femininity. Women said:

“*It looked like hair that never grew (…), but eventually it did*”

(Cod. 5.126).

## 4. Discussion

This study aims to explore the experiences of women with leukemia along the disease’s trajectory, from diagnosis to treatment and follow-up after stem cell transplantation (SCT).

To the best of our knowledge, this is the first study to analyze in depth what women, and not indiscriminately the entire leukemia population, experience when being diagnosed with leukemia and going through all the necessary treatments to cope with the disease, including the post-transplant follow-ups. The research was elaborated according to the GT method and using the framework of female gender and intersectionality [59].

In the logic of intersectionality and female gender, the model developed takes into account the intersections between illness and the physical, psychological, and social dimensions of women, showing a diverse and complex whole, which is the experience of these women [60].

‘Inner strength’ and resilience in women

The model developed from the analyzed data highlights an important first aspect that gave the theory its name: women with leukemia, between inner strength and the fight against prejudice.

The ‘core category’ identified in the ‘women’s inner strength in the face of the entire illness trajectory’ is a strength that some women recognize they possessed as their characteristic even before the illness, which in others interviewed was instead recognized, even with a certain astonishment, as a positive element arising precisely during the illness [61]. Interestingly, in most cases, the women acquire this awareness precisely at the time of follow-up, through personal retrospective reflection on what has happened to them and on their sometimes not-taken-for-granted abilities to support and cope with the illness [62].

In our results, we conceptualized resilience and strength. Women never speak of resilience but of strength, in agreement with Tan’s study [63], which concludes that cancer patients are rarely mentioned as using the term resilience directly, perhaps because it is more of a vocabulary term for health professionals.

Another characteristic element of women is their ability to fight in various ways, whether they activate mechanisms of active struggle against the disease, or whether they allow themselves to go through this new challenge in their life’s journey, with only seeming surrender. In this dialectic of resistance and surrender, women build a strong and lasting relationship with the disease, not by exorcising it, suffering it, or fearing it, but by collaborating with it. That is, they realize that it is only by collaborating with the disease, as hard, long, or unpredictable its course, that women manage to create a kind of alliance with it—not confrontation, not subjection, not a taboo to be shunned with fear, but a relationship that allows women to mold the disease on themselves.

Body image and ‘diminished beauty’

According to Brunet [33], women perceive a completely altered image of themselves due to the disease, but they are also able to identify intrapersonal and interpersonal factors to deal with this problem. Among the intrapersonal factors, the women in our study highlight that their inner strength, the ability to adapt to changes and find positivity even in the ‘drama of the disease’, are elements that promote acceptance of what is happening to them.

Among the interpersonal factors, relationships with family, support from family members, the sociability of the interviewees, and support from friends play an important role in the illness path.

The women also try to remain ‘beautiful’ despite the drama of their illness and this can be read as a constant and personal struggle against prejudice and stereotypes. The women perceive that the outside world has a particular gaze on them, perhaps judging and devaluing them, in the sense of highlighting the difference the illness has caused in their body and identity. The women interviewed state that they feel the eyes of others on them; we do not know how much these eyes are judgmental towards them or how much it is just the women’s perception.

Kang’s study [31] correlates the change in body image during the transplant phase with low quality of life and depression and asks, in conclusion, what the body image would be like after the transplant. In our study, the positivity towards life and the sense of hope for the future that these women experience after the transplantation is highlighted, albeit not in overt expressions.

Family relationships and new relations

The dimension of relationships has, in this study, two different connotations, one that is also frequently found in several other studies on cancer patients that highlight the experiences experienced, but also a very particular connotation, relating to the fact that women know how to be surprised by even unexpected elements of relationships [62]. It is known from the literature that marital relationships are severely tested by oncological disease, that other very solid relationships can fray and eventually disappear, with a great feeling of suffering and loneliness on the part of the patients. In our study, women take comfort in relationships that remain solid (with their husband, mother, or children), but they also know how to be satisfied with new relationships and friendships that can still provide significant support such as relationships with professionals [64].

The category of living in the present is one of the dimensions most present in the oncology literature in relation to people’s experiences of illness. The element of moving forward is closely related to what has been described above concerning women’s inner strength and their ability to fight. The awareness, in women, that there is no going back is recognized early on; however, not being able to go back is not experienced as negativity, but is a precursor to the strong decision to live with the illness and to move forward.

In this intersectional view, the experience of leukemia in women is not only about the disease but also about how it interacts with social norms concerning beauty, care, and emotional strength, creating a distinct and multifaceted experience.

### 4.1. Limitations

The main limitations of our study can be summarized as follows. The research environment was a hospital hematology department. This sample was limited to women who received treatment in this specific setting. The patients were interviewed during the follow-up phase when they were in the ward. Although we examined the women’s experience along the entire care pathway with the interviews, no specific clinical data were collected.

All women were interviewed after the successful transplantation. Their retrospective recollection of the disease course may have been altered by the long period of time since diagnosis.

The participants represent a small number of white women, of middle age, from a specific region in Italy. They are all educated, so they are capable of a certain degree of reflection. Other research could be on groups of women of different ages (younger or older) or with other social and ethnic characteristics.

### 4.2. Implications for Practice and Research

The results of our study may have implications for care practice by reinforcing the importance of using the bio-psycho-social model in care and treatment. The in-depth analysis of illness experiences revealed aspects that are not easily understood through a purely biological logic of analysis and care. The study suggests that professionals should pay attention to human relationships for the comfort of the sick person, as well as leave room for the personal resources of sick people and their relatives, which are fundamental to managing the illness and adding quality to life. The holistic view of the person, encompassing states of mind, inner strength, coping skills, relationship skills, positive reaction to changes, and strength to react to prejudices could be used for more personalized care and care that takes into account not only biological reactions but also psycho-social reactions.

Future gender-related research could provide innovative elements of care, reduction of prejudice, and equity of access to care.

## 5. Conclusions

Using the methodology of grounded theory and an intersectionality approach, the research team identified a model they called ‘Women with leukemia, between inner strength and fighting prejudice’. This model highlights the strength that the interviewees said they had during their illness, namely, their ability to ‘befriend’ the disease in order to live with it better, activating inner and outer resources that perhaps the women themselves did not know they had.

Further research is crucial to fully comprehend these psycho-social processes and to unearth gender differences that, once revealed, could be harnessed in order to enhance the quality of life of patients and families throughout the illness trajectory. This potential for further research offers hope and optimism for the future of patient care.

## Figures and Tables

**Table 1:** Criteria for inclusion and exclusion of study participants.

Inclusion Criteria	Exclusion Criteria
(i) Female gender. (ii) Diagnosis of leukemia at post-transplant follow-up. (iii) Age between 25 and 55 years. (iv) Fluency in Italian language. (v) Consent to participate in the research.	(i) Male gender. (ii) Not having completed treatment up to the post-transplant stage. (iii) Have other oncohematological diseases.

**Table 2:** Semistructured interview outline.

1. Opening question, to encourage free communication by the respondent.
2. Follow-up questions to better understand the process concerning the following issues: (i) the narrative of the experience of illness as a whole; (ii) evolution and changes that occurred during the illness process; (iii) what facilitated/hindered the quality of life during the prolonged illness journey.
3. Closing question, with thanks and regards.

**Table 3:** Characteristics of data coding.

Codes	Description
Open Coding	The research team started coding all the data in every possible way. The consequence of this open coding was a multitude of descriptions of possible concepts that often did not fit the emerging theory. In this phase, the researcher used a constant comparative method for open coding and the coding of qualitative data line by line. This phase aimed to illustrate the emerging concept and its properties. Several meetings were conducted between the authors until a consensus on the codes was reached, and a codebook with the first identification of 160 codes (in vivo codes) was produced [49].
Axial coding	This procedure extended the initial stage of open coding by coding 195 codes, systematically exploring the relationships between categories and subcategories through a coding paradigm to establish distinctions and find similarities and differences. This process laid the foundation for selective coding and identifying a central category of the phenomenon studied, which integrated the categories and led to theory generation [45,52]. The core category was chosen through several team meetings. This category was central and connected to all the others, recurred frequently in the data, and possessed significant explanatory power.
Theoretical coding	An explanatory theoretical model of the studied phenomenon was constructed, in line with the research question formulated in abstract terms, and the relationships between the identified categories were identified [53].

**Table 4:** Grounded theory’s essential data processing elements.

Data Processing Elements	Description
(i) Constant comparison	Data were continuously compared with each other on more and more abstract levels to further develop categories, concepts, and theory [57,58].
(ii) Coding	Data analysis involved applying descriptive labels to data or datasets for analytical purposes. It organized the data at increasingly higher levels, moving from codes to categories, from concepts to theory.
(iii) Memoing processing	The research team wrote memos and compared them to move from themes to concepts.

**Table 5:** Main characteristics of participants during their treatment journey.

Variables	Descriptives
Age (years) M ± SD	46.5 (±7.8)
Gender (female) M ± SD	13 (100)
Married (N. %)	13 (100)
White (N. %)	13 (100)
Nationality: Italian (N. %)	13 (100)
Education	
High School (N. %)	7 (53.8)
University study (N. %)	6 (46.2)
National Healthcare Service	
Free health, psychological, and social services (N. %) History of mental health problems	13 (100) 0 (00)
Typology of cancer	
AML = Acute Myeloid Leukemia (N. %)	7 (53.8)
ALL = Acute Lymphoblastic Leukemia (N. %)	5 (38.5)
MDS = Myelodysplastic Syndrome (N. %)	1 (7.7)
Treatments	
Chemotherapy (N. %)	13 (100)
Hematopoietic Stem Cell Transplantation (HSCT) (N. %)	13 (100)
Treatment-related complication experienced during first 100 days post HSCT	
Febrile neutropenia (FN) (N. %)	13 (100)
Oral Mucositis (OM)(N. %)	10 (76.9)
Acute Graft versus Host Disease (aGvHD) (N. %)	2 (15.4)
Chemotherapy Induced Nausea and Vomiting (CINV) (N. %)	7 (53.8)
Hospital stay during Stem Cell Transplantation (days) M ± SD	41.5 (±17.1)
Time between diagnosis and Stem Cell Transplantation (days) M ± SD	917.5 (±535.4)
Days from HSCT to Interview M ± SD	638.2 (±446.1)

**Table 6:** Identification of the main concepts that emerged, definitions, and reference codes.

Concepts	Definition	Verbatim/Codes
Strength	The women recognize that they ‘are strong’ and have even gained strength from the disease despite alternating moments of fragility with moments of discovering positive elements that have been helpful.	‘*I appreciate the strength, the courage I had*’ (Int 3). ‘*There was the family, it was the baby, my husband because I am the backbone, I mean, I always was, even when I was inside hospitalised*’ (Int 7). “*From the hospital it was always me giving strength to those at home*” (Int. 9)
Drama	The moment of diagnosis is experienced as sudden and unexpected. In relation to future uncertainty, moments of doubt alternate with moments of great despondency.	‘*There was an initial phase of… ‘Oh my God, drama’; it’s OK, but also ‘whatever I do now*’ (Int. 4). ‘*I mean, little by little, this thing turned me off, let us say, it took my strength away*’ (Int. 2).
Fight	The fight becomes a constant element in women, whether they express it clearly or show themselves as surrendering, they are always inwardly in a state of showing resistance (more or less active) to the disease.	‘*That evening, I knew what I had and had to do to cure myself. And so I did*’ (Int 6).‘*I said I cannot do that from this thing here, which is staying at home sick and waiting to heal and recover*’ (Int 7). One perceives a condition of constant struggle, sometimes revealed in the firm will, sometimes in an apparent ‘following the wave’ (Memo).
Relationship	During illness, women ‘rediscover’ important relationships and affections, be they family, friends, or the care team.	*So the affections in this sense have come out, and so it is something good that has been left for me by the illness’* (Int. 6).‘*Connecting maybe could be something that helps to get strength together*’ (Int. 11). “*My mother and brother were also very close to me..then my brother also donated marrow to me*” (Int. 10). ‘*In here, you are cared for, looked after, cuddled, extra-cuddled because they are wonderful here*’ (Int. 3).
Changes	Women find themselves at the mercy of various changes, which touch and upset certainties. These are very often physical changes, such as body image, but also changes of character or lifestyle, involving the deepest part of themselves.	“*But in the face idea that it changes a lot afterwards, some things you probably cannot avoid, that is the hair. I mean now I do not know how much you can do*” (Int 5). “*I was sick, I had lost 20 kilos, I could hardly stand up, and then I just collapsed, a bit of depression*” (Int. 9) “*Because, unfortunately, the illness has left some marks on me, the tiredness you feel three times as much, partly because of your age and partly because of everything I have been through…*”. (Int 1).
Resilience	Using all their resources (internal and external), women can adapt to illness situations.	“*Then I did not ask myself so many questions, I did not ask myself where I was going to end up following the path…and I followed the path… that is it….*” (Int. 6).“*Leukemia is a lady who entered inside me…. I do not want to fight it now, but I want to collaborate because she must understand that this is not her place. After all, I have so many other things to do!* ” (Int 10).

## Data Availability

The datasets analyzed during the current study are available from the corresponding author upon reasonable request.
